# Clinical, retrospective case-control study on the mechanics of obstacle in mouth opening and malocclusion in patients with maxillofacial fractures

**DOI:** 10.1038/s41598-018-25519-0

**Published:** 2018-05-16

**Authors:** Hai-Hua Zhou, Kun Lv, Rong-Tao Yang, Zhi Li, Xue-Wen Yang, Zu-Bing Li

**Affiliations:** 1 The State Key Laboratory Breeding Base of Basic Science of Stomatology (Hubei-MOST) & Key Laboratory of Oral Biomedicine Ministry of Education, School & Hospital of Stomatology, Wuhan University, Wuhan, People’s Republic of China; 2 Department of Oral and Maxillofacial Surgery, College and Hospital of Stomatology, Wuhan University, Luo yu Road, 237#, Wuhan, 430079 Hubei People’s Republic of China

## Abstract

This study aims to identify and distinguish various factors that may influence the clinical symptoms (limited mouth opening and malocclusion) in patients with maxillofacial fractures. From January 2000 to December 2009, 963 patients with maxillofacial fractures were enrolled in this statistical study to aid in evaluating the association between various risk factors and clinical symptoms. Patients with fractured posterior mandibles tended to experience serious limitation in mouth opening. Patients who sustained coronoid fractures have the highest risk of serious limitation in mouth opening (OR = 9.849), followed by arch fractures, maxilla fractures, condylar fractures, zygomatic complex fractures and symphysis fractures. Meanwhile, the combined fracture of zygomatic arch and condylar process results in normal or mild mouth opening. High risks of sustaining malocclusion are preceded by the fracture of nasal bone (OR = 3.067), mandible, condylar neck/base, combined fracture of zygomatic arch and condylar process, mandibular body, bilateral condylar, dental trauma, mandibular ramus, symphysis, mandibular angle and mid-facial. Patients who experienced serious limitation in mouth opening are treated with surgery more frequently (OR = 2.118). No relationship exists between the treatment options and the patients with malocclusion.

## Introduction

The primary goals in treating maxillofacial fractures are to establish and maintain normal occlusion and attain the preinjury mobility and function of the jaws and the preinjury 3-dimensional (3D) facial contours^1,2^. Numerous studies have been conducted on the epidemiology and treatment of maxillofacial fractures. However, works on the basic mechanism of clinical symptoms, such as limited mouth opening and malocclusion, are limited; for instance, why different types of maxillofacial fractures display different symptoms in mouth opening and occlusion. In clinical practice, on the basis of experience alone, the occurrence and type of maxillofacial fractures are difficult to determine. More seriously, maxillofacial fractures are difficult to treat effectively and accurately^3^. Accordingly, postponed or inappropriate intervention may lead to the malformation of osseous callus and soft tissue fibrosis^4^, and even the occurrence of facial deformity (facial depression^5^, temporomandibular joint ankylosis and micrognathia^6^).

In previous studies, we analysed the mechanisms in the production of mandibular fractures^5,7–10^ and evaluated various factors that are correlated with maxillofacial fractures^11–16^. In the present study, we attach more importance to the symptoms of patients who sustained maxillofacial fractures. The exploration and analysis of the mechanism of the clinical symptoms of maxillofacial fractures could provide an in-depth understanding of the mechanism of maxillofacial fractures for an accurate and effective assessment of the patients’ condition and treatment of maxillofacial fractures, while reducing the individual, family, social and national burden and promoting the recovery of the patients’ daily life [communication (speech and facial expression), nutrition, breathing, hearing, vision and cosmetic consequences]^17^.

## Materials and Methods

### Ethics Statement

We conducted a hospital-based retrospective case-control study at Stomatology College and Hospital, Wuhan University, from January 2000 to December 2009. The protocol and the survey and consent forms were approved by the Institutional Review Board (IRB) of Wuhan University (approval number: 2018-B05). The written consents provided by the patients were waived by the approving IRB.

### Patient Population and Data Collection

This study included patients with maxillofacial fractures who were admitted at their initial visit to the Department of Oral and Maxillofacial Surgery, Stomatology College and Hospital, Wuhan University, from January 2000 to December 2009. Patients were excluded as study subjects based on the following: (1) Repeated admissions, (2) Incomplete information, (3) Maxillofacial fractures previously treated surgically in other hospitals. Data on age, sex, trauma etiologies, soft tissue injuries, dental trauma, general injuries (including traumatic head injuries and ocular trauma), malocclusion, maxillofacial fracture type, treatment delay times and treatment methods were collected and standardized by an investigator based on the patients’ case histories, clinical and radiographic examinations and medical records.

The injury mechanisms were classified as assault, road traffic accident (motor vehicle accident (MVA), motorcycle accident and bicycle accident), fall (at ground or high levels), sports- or work-related accident and others.

Mandibular fractures were classified as condylar (condylar head/comminuted, condylar neck, condylar base fractures), symphysis, body, angle, ramus, coronoid and alveolar fractures. Mid-facial fractures included zygomatic complex fracture (ZCF), zygomatic arch, Le Fort (I/II/III), nasal, orbital, maxilla and alveolar fractures.

Facial fractures were categorized as multiple or single mid-facial fractures (zygomatic arch, zygomatic complex, orbital, maxilla and upper alveolar fractures), combined fractures of the mid-face and mandible, multiple and single mandibular fractures, unilateral/bilateral condylar fractures, combined fractures of the condyle and zygomatic arch.

Critical clinical symptoms included limited mouth opening and malocclusion (inadequate restoration of occlusal relationships/persistent occlusal change^18^, including anterior open bite, lateral open bite, cross bite, mandibular retrognathia, maxillary retrognathia or laterognathia^19^, etc.). Soft tissue and/or dental injuries in the maxillofacial area were recorded. Associated fractures, such as skull, ocular, thoracic, cervical, vertebra, pelvis, extremity and abdominal injuries, were also documented as ‘other body fractures/injuries.’

The maximal mouth opening were measured with a ruler graded in millimeters according to Agerberg^20^ and Obwegeser *et al*.^21^. The patients were divided into the normal (opening > 3 cm), minor (2 cm < opening ≤ 3 cm), moderate (1 cm < opening ≤ 2 cm), severe (0.5 cm < opening ≤ 1 cm) and serious (opening ≤ 0.5 cm) groups.

### Case and Control Groups

Cohort study 1: Patients diagnosed with limited mouth opening comprised the case group. Meanwhile, patients without limited mouth opening comprised the control group.

Cohort study 2: Patients diagnosed with malocclusion comprised the case group. Meanwhile, patients without malocclusion comprised the control group.

### Statistical Analysis

Statistical analysis was performed using the SPSS software (version 19.0; SPSS, Chicago, IL, USA). Continuous variables were reported as mean ± SD and assessed using independent sample t-tests as necessary. The chi-square test was used to compare the categorical variables. Fisher’s exact test was utilized when observation in any cell of the 2 × 2 table was expected to be less than five. Odds ratio (OR) and 95% confidence interval (CI) were used to assess the risk of patients who sustained limited mouth opening or malocclusion. The risk factors of limited mouth opening were further analysed by using ordinal logistic regression. Logistic regression analysis was utilized to control the confounding variables. Probabilities of P < 0.05 were considered significantly different.

### Results

The risks of patients who sustained serious limited mouth opening according to various variables are summarized in Table 1 (ordinal logistic regression). Patients who sustained fractures of the posterior mandible tended to be associated with serious limitation in mouth opening (coronoid fracture: OR = 2.989; condylar fracture: OR = 2.370; ramus fracture: OR = 1.592), compared with patients who sustained fractures of the anterior mandible (alveolar fracture: OR = 1.567; symphysis fracture: OR = 1.297; body fracture: OR = 1.225), except angle fractures (OR = 1.249) and condylar head fractures (OR = 1.206).

**Table 1:** Evaluation of risk factors in patients with limited mouth opening by ordinal logistic regression.

Factors		Case	Control	*p*	OR	95% C.I.	
						Lower	Upper
Threshold							
y = 1		44	919	0.000	0.059	0.017	0.204
y = 2		252	711	0.420	0.610	0.183	2.032
y = 3		436	527	0.009	4.968	1.484	16.643
y = 4		51	912	0.000	30.054	8.732	103.338
Location							
Malocclusion	x1	601	362	0.732	0.952	0.725	1.251
Mandibular fractures	x2	760	203	0.572	1.332	0.493	3.600
Single mandibular fracture	x3	332	631	0.346	1.293	0.757	2.208
Multiple mandibular fractures	x4	428	535	NA	NA	NA	NA
Condylar fractures	x5	471	492	0.009	2.370	1.246	4.509
Condylar head fractures/comminuted fractures	x6	243	720	0.420	1.206	0.765	1.900
Condylar neck/base fractures	x7	261	702	0.030	0.612	0.393	0.953
Bilateral condylar fractures	x8	168	795	0.177	0.742	0.481	1.145
Unilateral condylar fracture	x9	303	660	NA	NA	NA	NA
Mandibular angle fracture	x10	116	847	0.437	1.249	0.713	2.184
Mandibular body fracture	x11	178	785	0.429	1.225	0.741	2.026
Symphysis fracture	x12	347	616	0.261	1.297	0.824	2.042
Alveolar fracture in mandible	x13	11	952	0.457	1.567	0.480	5.114
mandibular ramus fracture	x14	13	950	0.403	1.592	0.535	4.740
Coronoid fracture	x15	22	941	0.020	2.989	1.190	7.508
Mid-facial fractures	x16	375	588	0.067	0.478	0.217	1.052
Single mid-facial fractures	x17	95	868	0.244	1.467	0.770	2.795
Multiple mid-facial fractures	x18	280	683	NA	NA	NA	NA
Zygomatic arch fracture	x19	237	726	0.001	2.312	1.425	3.747
Zygomatic complex fractures	x20	267	696	0.304	1.344	0.765	2.363
Maxilla fracture	x21	122	841	0.087	1.508	0.942	2.416
Alveolar fracture in upper jaw	x22	31	932	0.741	1.133	0.541	2.370
Orbital fracture	x23	128	835	0.857	1.042	0.666	1.629
Nasal fracture	x24	42	921	0.472	1.288	0.645	2.570
Lefort I/II/III fractures	x25	36	927	0.405	0.726	0.342	1.543
Fracture of mid-facial and mandible	x26	172	791	NA	NA	NA	NA
Fracture of condyle and zygomatic arch	x27	32	931	0.170	0.558	0.242	1.284
Dental injuries	x28	409	554	0.275	0.863	0.663	1.124
Other body fractures/injuries (including traumatic head injuries or ocular injuries)	x29	170	793	0.031	1.559	1.042	2.333
Associated with traumatic head injuries	x30	76	887	0.731	0.908	0.524	1.575
Associated with traumatic ocular injuries	x31	170	793	0.038	1.523	1.024	2.266
Soft tissue injuries	x32	739	224	0.279	1.182	0.874	1.597
Surgical treatment	x33	927	36	0.423	0.770	0.406	1.459
Sex	x34	753	210	0.673	0.938	0.698	1.262
Age	x35			0.519	0.997	0.988	1.006
Treatment delay time	x36			0.002	0.993	0.989	0.997
Assault related accidents	x37	128	835	0.647	0.825	0.362	1.878
Bicycle accidents	x38	59	904	0.369	0.658	0.264	1.637
MVA	x39	297	666	0.966	0.983	0.441	2.190
Fall at ground level	x40	129	834	0.261	0.617	0.266	1.430
Fall from high	x41	108	855	0.662	1.212	0.511	2.872
Motorcycle	x42	151	812	0.694	0.847	0.371	1.935
Other	x43	48	915	0.281	0.599	0.236	1.519
Sport related accidents	x44	19	944	0.805	1.156	0.366	3.651
Work related accidents	x45	24	939	NA	NA	NA	NA

Y = 1: normal group (opening > 3 cm);

Y = 2: minor group (2 cm < opening ≤ 3 cm);

Y = 3: moderate group (1 cm < opening ≤ 2 cm);

Y = 4: severe group (0.5 cm < opening ≤ 1 cm);

NA: not application;

MVA: motor vehicle accidents.

The relationship between various etiologies and the risk of limited mouth opening is further summarized in Table 2. Patients who sustained coronoid fractures had the highest risk of serious limitation in mouth opening (OR = 9.849), followed by arch fractures (OR = 3.202), maxilla fractures (OR = 2.914), condylar fractures (OR = 2.764), ZCF fractures (OR = 2.701) and symphysis fractures (OR = 2.694). As far as traumatic etiologies, such as assault, bicycle and MVA, are concerned, most of the patients tended to sustain moderate mouth opening (1 cm < opening ≤ 2 cm, OR > 1) and less probability of serious limitation in mouth opening (opening ≤ 0.5 cm, OR < 1). Patients with condylar neck/base fractures were more prone to the occurrence of mild mouth opening (OR > 1) and less to the occurrence of severe or serious limitation in mouth opening (OR < 1).

**Table 2:** Multivariate logistic regression analysis of patients with limited mouth opening.

Factors		Serious group (opening ≤ 0.5 cm) OR	Severe group (0.5 cm < opening ≤ 1 cm) OR	Moderate group (1 cm < opening ≤ 2 cm) OR	Minor group (2 cm < opening ≤ 3 cm) OR	Normal group (opening > 3 cm OR
Malocclusion	x1	0.683	1.037	0.960	1.266	0.477
Mandibular fractures	x2	0.271	1.232	1.496	1.608	0.287
Single mandibular fracture	x3	1.520	1.281	0.969	0.826	0.434
Multiple mandibular fractures	x4	NA	NA	NA	NA	NA
Condylar fractures	x5	2.764	1.936	1.328	0.300	1.290
Condylar head fractures/comminuted fractures	x6	1.187	1.288	0.933	0.824	1.652
Condylar neck/base fractures	x7	0.363	0.589	1.282	1.379	1.705
Bilateral condylar fractures	x8	0.972	0.757	0.868	1.672	0.556
Unilateral condylar fracture	x9	NA	NA	NA	NA	NA
Mandibular angle fracture	x10	0.450	1.039	1.717	0.526	0.340
Mandibular body fracture	x11	1.163	1.197	1.075	0.747	0.907
Symphysis fracture	x12	2.694	1.046	1.031	0.759	0.680
Alveolar fracture in mandible	x13	—	1.141	2.192	0.500	—
Mandibular ramus fracture	x14	—	1.599	1.570	0.414	—
Coronoid fracture	x15	9.849	1.612	0.621	0.458	—
Mid-facial fractures	x16	0.072	0.843	1.050	2.042	1.033
Single mid-facial fractures	x17	2.070	1.304	1.179	0.382	2.527
Multiple mid-facial fractures	x18	NA	NA	NA	NA	NA
Zygomatic arch fracture	x19	3.202	1.461	1.455	0.404	0.719
Zygomatic complex fractures	x20	2.701	0.936	1.285	0.594	1.100
Maxilla fracture	x21	2.914	1.082	0.918	0.655	1.123
Alveolar fracture in upper jaw	x22	—	1.995	0.719	0.739	1.023
Orbital fracture	x23	0.888	1.099	1.084	0.767	1.604
Nasal fracture	x24	0.522	1.125	1.236	1.054	0.367
Lefort I/II/III fractures	x25	1.812	0.389	1.444	0.650	5.512
Fracture of mid-facial and mandible	x26	NA	NA	NA	NA	NA
Fracture of condyle and zygomatic arch	x27	1.218	0.908	0.387	2.956	2.020
Dental injuries	x28	0.885	0.925	0.904	1.118	1.339
Other body fractures/injuries (including traumatic head injuries or ocular injuries)	x29	2.054	1.386	1.013	0.674	0.640
Associated with traumatic head injuries	x30	1.271	1.108	0.526	1.713	1.260
Associated with traumatic ocular injuries	x31	2.085	1.753	0.624	1.156	0.478
Soft tissue injuries	x32	0.927	1.120	1.112	0.805	0.949
Surgical treatment	x33	2.118	0.520	1.093	1.553	0.703
Sex	x34	0.729	0.990	1.041	1.002	1.241
Age	x35	1.002	1.000	1.001	0.998	0.998
Treatment delay time	x36	1.014	1.004	1.004	0.996	0.987
Assault related accidents	x37	0.114	0.725	2.066	1.257	0.112
Bicycle accidents	x38	0.307	0.453	2.183	1.136	0.428
MVA	x39	0.394	0.657	2.328	0.834	0.225
Fall at ground level	x40	0.325	0.332	2.586	1.031	0.485
Fall from high	x41	0.491	0.569	3.178	0.682	0.047
Motorcycle	x42	0.184	0.491	3.451	0.806	0.153
Other	x43	0.142	0.513	1.589	1.989	0.166
Sport related accidents	x44	0.510	0.550	3.539	0.649	—
Work related accidents	x45	NA	NA	NA	NA	NA

Note: OR = odds ratio; 95% confidence interval was not listed.

Interestingly, fracture of the zygomatic arch or condylar process resulted in the high occurrence of serious limitation in mouth opening (OR > 1), whereas the combined fracture of zygomatic arch and condylar process were more prone to normal or mild mouth opening (OR by ordinal logistic regression: 0.558; OR by logistic regression analysis: OR_minor mouth opening_ = 2.956, OR_normal mouth opening_ = 2.020).

Accordingly, patients who sustained serious limitation in mouth opening were treated by surgery more frequently (OR = 2.118), whereas patients with normal mouth opening were frequently treated nonsurgically.

The risk of sustaining malocclusion in associated with different factors, which are summarized in Table 3. We found that nearly all traumatic factors had a high risk of occurrence of malocclusion (OR > 1), except for patients who sustained fractures of the condylar head/mandibular alveolar/zygomatic arch/coronoid process/maxilla alveolar/single mandible/single maxilla. The high risks of sustaining malocclusion in descending order are as follows: fracture of nasal bone (OR = 3.067), mandible (OR = 2.721), condylar neck/base (OR = 2.173), combined fracture of zygomatic arch and condylar process (OR = 1.846), mandibular body (OR = 1.731), bilateral condylar (OR = 1.595), dental trauma (OR = 1.564), mandibular ramus (OR = 1.432), symphysis (OR = 1.338), mandibular angle (OR = 1.330) and mid-facial (OR = 1.249). Interestingly, body injuries (including traumatic head and ocular injuries) resulted in the high occurrence of malocclusion (OR > 1). Heavy impact damages (motor vehicle accidents or motorcycle accidents) also resulted in the high occurrence of malocclusion (OR > 1). Interestingly, fractures of the zygomatic arch resulted in the low occurrence of malocclusion (OR = 0.452).

**Table 3:** Multivariate logistic regression analysis of patients with malocclusion.

Factors		Case (n = 601)	Control (n = 362)			95% C.I.	
				*p*	OR	Lower	Upper
Limited mouth opening	x1	1.86 ± 0.756	1.87 ± 0.861	0.847	0.981	0.808	1.192
Mandibular fractures	x2	530	230	0.159	2.721	0.675	10.971
Single mandibular fracture	x3	188	144	0.097	0.532	0.253	1.121
Multiple mandibular fractures	x4	NA	NA	NA	NA	NA	NA
Condylar fractures	x5	346	125	0.947	1.031	0.417	2.552
Condylar head fractures/comminuted fractures	x6	165	78	0.429	0.772	0.407	1.465
Condylar neck/base fractures	x7	210	51	0.013	2.173	1.180	4.002
Bilateral condylar fractures	x8	139	29	0.147	1.595	0.849	2.996
Unilateral condylar fracture	x9	NA	NA	NA	NA	NA	NA
Mandibular angle fracture	x10	80	36	0.470	1.330	0.614	2.885
Mandibular body fracture	x11	136	42	0.140	1.731	0.835	3.586
Symphysis fracture	x12	263	84	0.382	1.338	0.696	2.572
Alveolar fracture in mandible	x13	6	5	0.302	0.474	0.115	1.958
Mandibular ramus fracture	x14	11	2	0.672	1.432	0.271	7.580
Coronoid fracture	x15	9	13	0.329	0.557	0.172	1.805
Mid-facial fractures	x16	189	186	0.664	1.249	0.458	3.403
Single mid-facial fractures	x17	47	48	0.886	0.942	0.419	2.119
Multiple mid-facial fractures	x18	NA	NA	NA	NA	NA	NA
Zygomatic arch fracture	x19	95	142	0.007	0.452	0.255	0.803
Zygomatic complex fractures	x20	133	134	0.894	1.051	0.509	2.167
Maxilla fracture	x21	70	52	0.784	1.086	0.603	1.955
Alveolar fracture in upper jaw	x22	17	14	0.695	0.833	0.334	2.078
Orbital fracture	x23	66	62	0.897	1.037	0.597	1.803
Nasal fracture	x24	30	12	0.012	3.067	1.284	7.329
Lefort I/II/III fractures	x25	24	12	0.639	1.253	0.489	3.209
Fracture of mid-facial and mandible	x26	NA	NA	NA	NA	NA	NA
Fracture of condyle and zygomatic arch	x27	25	7	0.280	1.846	0.607	5.616
Dental injuries	x28	293	116	0.008	1.564	1.122	2.181
Other body fractures/injuries (including traumatic head injuries or ocular injuries)	x29	124	46	0.067	1.650	0.965	2.823
Associated with traumatic head injuries	x30	51	25	0.988	0.995	0.488	2.027
Associated with traumatic ocular injuries	x31	79	91	0.168	0.708	0.433	1.157
Soft tissue injuries	x32	457	282	0.952	0.988	0.671	1.455
Surgical treatment	x33	580	347	0.978	1.011	0.459	2.228
Sex (man)	x34	478	275	0.163	1.306	0.898	1.899
Age	x35	30.24 ± 13.09	33.19 ± 13.77	0.028	1.013	1.001	1.025
Treatment delay time	x36	19.70 ± 29.56	18.60 ± 30.24	0.918	1.000	0.994	1.005
Assault related accidents	x37	71	57	0.873	1.085	0.400	2.945
Bicycle accidents	x38	33	26	0.774	0.849	0.278	2.596
MVA	x39	193	104	0.398	1.529	0.571	4.089
Fall at ground level	x40	76	53	0.927	0.953	0.341	2.662
Fall from high	x41	82	26	0.949	0.966	0.332	2.814
Motorcycle	x42	97	54	0.375	1.583	0.574	4.367
Other	x43	21	27	0.302	0.550	0.177	1.709
Sport related accidents	x44	12	7	0.635	0.715	0.179	2.857
Work related accidents	x45	NA	NA	NA	NA	NA	NA

No relationship exists between the treatment delay times and the clinical symptoms of limited mouth opening or malocclusion, regardless of the length of time that passed after the fracture of the upper and lower jaws (Tables 1 to 3, OR ≈ 1). No relationship exists between the treatment options and the patients with malocclusion (Table 3, OR = 1.011).

### Discussion

The movement of the mandible (mouth opening and closing) is controlled by several muscles that are attached to it. The masticatory muscles (from the zygomatic arch, inserted to the lateral aspect of ramus), temporalis (from the temporal lines of the parietal bone, inserted to the coronoid process and anterosuperior border of ramus) and medial pterygoid (from the pterygoid process and tuberosity, inserted to the medial aspect of ramus) control the elevation of mandible, while the medial and lateral pterygoids (from the pterygoid process, pyramidal process of palatine bone and tuberosity, inserted to the condyle and anteromedial part of the disk) control the protrusion and laterotrusion of the mandible. As the antagonism mechanisms, the temporalis controls the mandible retrusion, while the suprahyoid musculature controls the mandible decline^22^. However, the occurrence of maxillary or mandible fracture (or injury) leads to broken muscle balance and the change in the movement mechanism of the mandible. A comprehensive understanding of the various factors that influence the movement of mandible after maxillofacial trauma is important in providing clinical and research data for the effective management of these injuries.

The fracture of condylar process results in high risk of serious limitation in mouth opening. However, further study revealed that the condylar head leads to the high occurrence of serious or severe limitation in mouth opening, whereas condylar neck/base fracture does not affect mouth opening. These phenomena may be attributed to several reasons. The articular disk is attached to the medial and lateral poles of the condylar process, ensuring the synchronized movements of the condyle and disk during gliding movements^22^; however, in patients with condylar head fractures, the continued traction of the lateral pterygoid muscle results in the anteromedial displacement of the fragment in condylar head fractures^23^, which may impede the movement of the condylar head^8^. Condylar neck/base fractures destroy the integrity of the mandible, and the muscle tension of the articular disk and lateral pterygoid cannot perform the function of mandibular movement. Consequently, the gliding movements, protrusion and laterotrusion movement of the mandible are weakened. However, the descending movement of the mandible that is controlled by the suprahyoid musculature remains, and mouth opening is recovered with the weakening of edema and muscle spasms. Interestingly, the present study revealed that patients with bilateral condylar fractures are more prone to the occurrence of mild mouth opening. We propose that mandible movement that is centred by the mandibular foramen (a fulcrum, controlled by sphenomandibular ligament) is retained despite of the disappearance of the temporomandibular joint activity.

Patients who sustained coronoid or zygomatic arch fractures are the most prone to serious limitation in mouth opening. Coronoid fractures seem highly co-related to zygomatic arch fractures. Our previous study revealed that majority of the patients who sustained coronoid fractures also had fractured zygomatic archs (20 of 25 patients, 80%). Furthermore, nearly all patients (23 of 25 patients, 92%) with coronoid fractures showed limited mouth opening^5^. We propose that the fracture of the zygomatic arch or coronoid processes is usually attributed to the muscle compression or mechanical barrier in the process of mouth opening and closing, thus leading to the serious limitation in mouth opening. The masticatory muscles and temporalis are squeezed by the fracture of the zygomatic arch or coronoid process, resulting in reduced or dysfunctional muscle in the traction and shrinking process. In our experience, coronoid fracture removal is usually conducted when limited mouth opening cannot be resolved after the open reduction of maxillofacial fractures^5^.

The fracture of mandible seems more prone to the limited mouth opening than mid-facial fractures, except for the fracture of the zygomatic arch. Additionally, patients with fractured posterior mandibles are prone to serious limited mouth opening compared with the patients with fractured anterior mandibles. This is understandable because most of the muscles (masticatory muscles, temporalis, medial pterygoid and lateral pterygoid) control the movement of the mandible attached to the posterior mandible. Interestingly, for patients who sustained serious limited mouth opening are concerned, the possibility of surgical treatment is high (OR = 2.118). The risk of surgical treatment dropped to 0.703-fold for patients with normal mouth opening. This phenomenon explains the main purpose of surgical treatment is to recover normal mouth opening. Thus, in the present study, surgical treatment is considered for patients with fractured mandibles compared to patients with mid-facial fractures, especially for patients with fractured posterior mandibles.

Malocclusion common in patients with fractured mandibles, except those with fractured coronoid process/condylar head/mandibular alveolar. Patients with mid-facial fractures have no obvious relationship with malocclusion. This is not surprising because the porous structure of the mid-face lowers the risk of malocclusion in patients with sustained mid-facial fractures (OR_zygomatic arch_ = 0.452, OR_ZCF_ = 1.051, OR_maxilla_ = 1.086, OR_orbital_ = 1.037 and OR_Lefort_ = 1.253). In clinical practice, we rarely find situations where the dental arch is divided into several sections in the upper jaws of patients with mid-facial fractures. Patients who sustained heavy impact damage (from motor vehicle accidents or motorcycle accidents) are more prone to malocclusion; however, no relationship exists between the heavy impact damage and the limitation in mouth opening. Interestingly, patients with other body fractures/injuries (including traumatic head injuries or ocular injuries) are more prone to both malocclusion and serious limited mouth opening.

Patients with different fracture levels of the mandibular condylar process display different clinical symptoms. Patients with condylar base/neck fractures are more prone to malocclusion than to limited mouth opening. In contrast, patients with condylar head fractures show high occurrence of limited mouth opening than of malocclusion. In the present study, we found that patients who sustained serious limited mouth opening were treated by surgery more frequently (OR > 1), whereas patients with normal mouth opening were more frequently treated by nonsurgical approaches. Accordingly, the surgical treatment of condylar head fractures are considered more frequently than before. In fact, increasing studies are reporting good results and advantages of the surgical treatment of condylar head fractures^23–25^. In our past experience, the majority of condylar head fractures were treated by surgical procedures^8^. For patients with linear condylar base/neck fractures (nondisplaced or mild displacement), intermaxillary traction (non-surgical treatment) was considered first; surgical procedure was considered once the malocclusion could not be resolved by intermaxillary traction or if patients sustained serious dislocated fractures of the condylar base/neck.

In the present study, we found that no relationship exists between the treatment delay time (old or new fracture) and the clinical symptoms of limited mouth opening or malocclusion. Thus, early intervention of the patients with symptoms of limited mouth opening or malocclusion is important, patients should not depend on self-recovery or automatic healing. A surgical treatment plan should be considered for patients who sustained serious limited mouth opening. For instance, patients with fractured coronoid process/zygomatic arch/maxilla/condyle/ZCF/symphysis/Lefort/combined body injuries (including traumatic head injuries or ocular injuries) (Table 2) and simultaneously associated with serious limited mouth opening should consider surgical treatment first. Patients who sustained only malocclusion could consider a non-surgical treatment procedure (intermaxillary traction and/or fixation of loose teeth).

We acknowledge some flaws in our study. As a retrospective clinical case-control study, the incomplete information collected results in part loss of sample subjects. Information were collected based on case histories, and thus the reliability of information is dependent on how accurately the patients provided the information and the standard uniform recording by different physicians. Additionally, we should point out that some kinds of malocclusion are present in populations without facial fractures, we also don’t know if they had these malocclusion before the fractures occurred. However, a large sample size (963 patients) over a long period (10 years) may partially compensate for the above shortcomings. Regardless of this study’s results, a prospective, multicentre/multilevel and large sample study should be conducted in the future.

The present study revealed that patients associated with serious limited mouth opening should prefer surgical procedure. Patients who sustained only malocclusion or acceptable/normal mouth opening may consider a non-surgical treatment procedure; nonetheless, the patient’s appearance (3-dimensional facial contours) is also an important factor that needs to be considered. The accurate assessment of patients’ condition are helpful in the timely, correct and effective treatment of the their disease, while reducing the individual, family, social and national burden. Such a retrospective study can be used to guide the future funding of public health programs that are geared towards the prevention and treatment of such injuries.
